# Antecedents of Public Mental Health During the COVID-19 Pandemic: Mediation of Pandemic-Related Knowledge and Self-Efficacy and Moderation of Risk Level

**DOI:** 10.3389/fpsyt.2020.567119

**Published:** 2020-11-12

**Authors:** Shengnan Wang, Kai Feng, Ying Zhang, Jianan Liu, Wei Wang, Yongxin Li

**Affiliations:** ^1^Department of Psychology, Institute of Psychology and Behaviour, Henan University, Kaifeng, China; ^2^School of Management and Economics, North China University of Water Resources and Electric Power, Zhengzhou, China; ^3^Psychological Health Education Centre, Henan University of Animal Husbandry and Economy, Zhengzhou, China; ^4^School of Economics and Trade, Henan University of Animal Husbandry and Economy, Zhengzhou, China

**Keywords:** COVID-19, mental health, pandemic knowledge, self-efficacy, risk level, family-based social support

## Abstract

**Background:** COVID-19 affects not only patients' physical health but also their mental health. For the general public, although their physical health may not be directly affected, their mental health may be affected by stress, anxiety, and social panic caused by COVID-19. Controlling the pandemic should focus on not only physical health but also mental health. For the general public, mental health is even more important, as good mental health at the individual level can form a positive social mentality conducive to pandemic prevention and control. Therefore, it is important to assess mental health during the pandemic, and analyze risk and protective factors.

**Methods:** A self-compiled COVID-19 Social Mentality Questionnaire was used to conduct an online survey. A total of 16,616 participants responded, with 13,511 valid questionnaires.

**Results:** Results showed that 10.7% of participants rated their mental health as “worse than usual” during the pandemic, and there were gender, age, and educational differences. Social support was positively correlated with pandemic-related knowledge and self-efficacy, and could indirectly predict mental health. Pandemic-related knowledge was positively correlated with self-efficacy and mental health, and risk level was negatively correlated with mental health. Hierarchical regression analysis showed that pandemic-related knowledge played a partial mediating role in the relationship between social support and self-efficacy, while self-efficacy played a complete mediating role in the relationship between social support and mental health. Logistic regression analysis showed that risk level moderated the relationship between self-efficacy and mental health.

**Conclusions:** Social support can increase pandemic-related knowledge, thus improving self-efficacy and maintaining/promoting mental health. High risk levels can undermine the role of self-efficacy in promoting mental health. Therefore, in the fight against the COVID-19, people need to support and cooperate with each other, to improve self-efficacy and reduce risk, thus maintaining and promoting mental health.

## Introduction

Coronavirus Disease 2019 (COVID-19) is an acute respiratory disease that is caused by a novel coronavirus (1). It is highly infectious, and mainly transmitted through droplets and close contact with others (2). The incubation period is usually 0–14 days, and the longest is 24 days (3). The mortality rate is about 5.22% in China (4). Currently, there is no specific drug treatment for this virus. On the early morning of 31 January, 2020, Beijing time, the World Health Organization (WHO) declared the COVID-19 outbreak to be a “public health emergency of international concern,” and the need for pandemic prevention and control became increasingly severe. On 29 February, the Director-General of WHO, Dr. Tedros, announced that the global risk level of COVID-19 had been raised from “high” to “very high,” the highest level, given the spread of COVID-19 in many countries, and the severity of the pandemic in some countries. By 13 September, 2020, more than 29 million cases had been confirmed, and over 926,900 deaths had been recorded worldwide (4).

As a new infectious disease, COVID-19 not only affects patients' physical health but also may negatively impact mental health, due to the unclear information about the virus's source, pathogenesis, high infectivity, and lack of specific drugs for treatment. In the study of the antecedent variables of mental health, scholars have expressed strong concern over stressful events (5–8). There are various types of stressful events, including disasters similar to the COVID-19 pandemic, and these are characterized by unpredictability, suddenness, rapid speed, and high-intensity stress. When individuals are under constant, excessive stress, they will experience adverse effects and threats to their physical and mental health (6). Several studies have found that stressful events are an important factor related to mental health (7, 8). For example, researchers found that stressful events can negatively predict the mental health of college students (8). Therefore, for the general public, even if their physical health is not directly affected by COVID-19, their mental health may be affected, due to such factors as individual stress and anxiety caused by the pandemic.

Thus, in preventing and controlling the spread of COVID-19, it is necessary to pay attention to public physical and mental health. Further, for the general public, mental health may be even more important, as good psychological health at the individual level can form a positive social mentality that is conducive to pandemic prevention and control. Therefore, it is particularly important to assess people's mental health during the pandemic period, especially various risks and protective factors and their action mechanisms.

As a stress theory, the conservation of resource theory holds that individuals have a tendency to strive to acquire, maintain, nurture, and protect their cherished resources (9). Therefore, both the potential resource loss threat and the actual resource loss will cause individual tension and stress (9, 10). In other words, both at the perceptual level and the objective level, the loss of existing resources and the failure to obtain new resources will trigger the individual's stress response, which will affect the individual's health. In the face of a stressor as significant as a pandemic, people often need to consume more resources to maintain their original and normal state. However, individuals have limited resources; therefore, on the one hand, they will use their key resources to cope with the stressful situation in the current environment; on the other hand, they will deal with the possible stressful situation in the future through the active construction and protection of their existing resource reserves (usually the way to obtain new resources).

**Figure 1:**
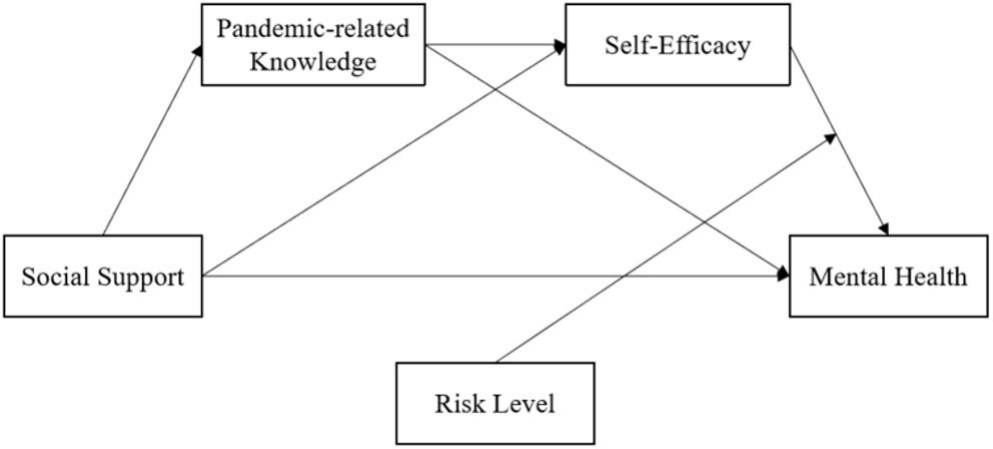
The conceptual model.

Hobfoll (9) believed that resources were the items that individuals thought valuable to them or the ways that could help them get valuable items, including Object resources, Conditions resources, Personal characteristics resources, and Energies resources. Specifically, the value of Object resources comes from their inherent physical properties or the individual identity information contained therein, such as houses, tools, etc. The value of Conditions resources derives from their positive significance for the future work and life of individuals, such as family and occupation. While the Personal characteristics resources refer to a variety of skills and characteristics possessed by an individual, such as self-efficacy, that are conducive to his/her resistance to pressure. And the value of Energies resources lies in their ability to help individuals acquire other resources they need, such as knowledge. In the COVID-19 pandemic, due to the influence of home quarantine order, the range of activities of individuals is limited and the object resources they have are relatively fixed and stable. Therefore, the resources that individuals can flexibly allocate are the conditions resources, personal characteristics resources, and energies resources they have, namely, family, occupation, self-efficacy, knowledge, and other resources. From the motivation of individuals to preserve and obtain resources, Halbesleben et al. (11) emphasized the subjective perception and appraisal of whether specific items contribute to the realization of their goals, and regardless of whether they actually contribute to the realization of goals. Thus, resources that are not normally considered of outstanding value may be of great significance to individuals in a particular situation. In a major pandemic, family members become a direct source of resources when people are under home quarantine order. In particular, those family members who are engaged in health care industry are not only the conditions resource of their family, but also the energies resource by sharing professional knowledge, so as to promote the accumulation of their family's personal characteristic resources and maintain mental health. However, a major pandemic cannot be resisted by a person or a family, and the effectiveness of its resource response is bound to be affected by external risks. Based on this, this study constructed a moderated mediation model with family-based social support as the independent variable, pandemic-related knowledge and self-efficacy as the mediating variable, and risk level as the moderating variable to investigate the impact of these variables on individual's mental health during the COVID-19 pandemic, as shown in Figure 1.

## Theory and Hypotheses

### Social Support and Mental Health

As an individual resource, social support includes mental and material support from various kinds of interpersonal relationships, including parents, other relatives, and friends. According to conservation of resources theory (9, 10), in a resource-losing context, the replenishment and increase of resources will be particularly important and more valuable to individuals. Which means, when an individual is under external pressure, his/her demand for resources will be more vigorous, and when new resources are injected at this time, the efficacy of new resources will be played to a greater extent. At the same time, according to effort-recovery theory, if an individual's consumed resources are not replenished in time, or if the replenishment is insufficient, his or her nervous system will remain active, making the individual unable to regain a state of self-equilibrium (12). However, a serious pandemic cannot be prevented by one person alone. Thus, despite using internal resources to cope with challenges, individuals will also use the external resource of social support to address current problems and threats.

According to the buffer model of social support, social support can provide individuals experiencing a state of stress with protection and exert a “buffer” effect to reduce individual adverse reactions (13, 14). Moreover, previous studies have shown that social support can effectively predict mental health (15, 16). Social support primarily includes two dimensions: objective support and subjective support (17). Previous studies have shown that in the action mechanism between mental health and social support, social support enables individuals to generate different views and corresponding emotions for certain objectives and events, and by perceiving and making use of these supports, individuals may change their attitudes toward life (17). Therefore, they could significantly reduce the negative impact of the objectives and events, and even obtain more satisfaction from the experience, thus naturally improving their mental health (18). Additionally, based on their findings, Xiao and Yang (13) proposed support utilization as the third dimension of social support. The results of a meta-analysis showed that subjective support and support utilization had positive effects on mental health, while objective support had a comparatively smaller positive effect on mental health (19). Besides, previous studies have confirmed that being lacking of social support may increase individuals' insomnia and suicide ideation during the COVID-19 pandemic (20–22). For example, Staines (20) and Killgore et al. (21) conducted an investigation on loneliness, suicide ideation, and insomnia of 1,013 English-speaking U.S. adults during the COVID-19 pandemic, in which it reported that 43% of the participants suffered loneliness and 56% of the participants had sleep difficulty, and consequently increased their mental health decline, and even triggered suicide ideation.

During the COVID-19 pandemic, as people are affected by home quarantine orders, family members have become important sources of social support, especially those with professional medical and nursing knowledge and skills. For individuals, this is not only an objective support, but also a strong perceived subjective support. Further, as it is convenient to acquire relevant knowledge and skills from health-care professional family members, this has a high level of support utilization. Therefore, social support was operationally defined in this study as family-based social support that whether individuals had family members in healthcare professions. Thus, Hypothesis 1 was proposed as follows: social support can positively predict individual mental health.

### Mediating Effects of Pandemic-Related Knowledge

Family members are important sources of social support, especially those with professional medical and nursing knowledge and skills. According to spillover theory, people tend to bring the knowledge, experience, emotions, skills, and behaviors they have constructed in the workplace into the home domain (23). A number of studies have also confirmed the existence of positive spillover. For example, Greenhaus and Powell (24) posited that instrumental paths and affective paths in work–family relationships can foster resource distribution from work to family, thus benefiting family members. Further, social support is an important driving factor for informal learning, which can promote knowledge-sharing (25), and thus increase individual knowledge. As a result, family members of healthcare workers may directly benefit from this, and have more access to pandemic-related knowledge than others. In a survey on public cognition of COVID-19 in China, researchers found that participants with healthcare workers in their families had more knowledge about COVID-19 and a higher level of cognition about the pandemic than the general public (26).

According to social cognition theory (27), indirect experience from others will affect the formation and development of individual self-efficacy. Pandemic-related knowledge gained from family members working in healthcare fields is a typical indirect experience that could, in theory, improve people's self-efficacy in dealing with the pandemic. Additionally, Lieberman (28) posited that children and adolescents can increase their knowledge by playing video games containing self-help and self-care skills, and improve their health decision-making ability, prevention efficacy, and self-rescue ability. In studies with adult participants, knowledge has been shown to mediate the association between social influences and self-efficacy in the prediction of health-related behaviors, such as eating habits (29). Ievers-Landis et al. (30) found that family social support for exercise could predict knowledge of physical activity designed to prevent osteoporosis. Therefore, Hypothesis 2 was proposed as follows: pandemic-related knowledge mediates the relationship between social support and self-efficacy.

### Mediating Effects of Self-Efficacy

Self-efficacy refers to the belief and confidence that an individual has in his or her ability to accomplish behavioral goals in a particular field (27). Bandura et al. (27) argued that individual cognition can have an impact on behavioral regulation, and self-efficacy, as a cognitive factor, is an important psychological motivator for maintaining individual self-regulation. More than that, in conservation of resources theory (9), self-efficacy is confirmed to be a typical resource of personal characteristics resources, which empowers individuals to accomplish tasks by adjusting their cognition of self-evaluation. Therefore, in this study, the operational definition of self-efficacy was individuals' self-efficacy to help themselves and others during the pandemic, which refers to an individuals' prediction of their success when they initiate self-help and help-seeking behaviors. It reflects an individual's confidence in being able to complete a behavior, and is the embodiment of individual self-efficacy in a specific situation. Studies have identified a positive correlation between self-efficacy and social support, and the stronger an individual's perception of social support, the higher his or her level of self-efficacy, and vice-versa (31). Freeman and Rees (32) found that the more external support athletes perceived, the more confident they would be during a competition. Yusoff (33) discovered that in stressful situations, social support from friends can have a comforting effect on individuals and help overseas students make positive mental adjustments. In a survey on the help-seeking efficacy of Chinese individuals during the COVID-19 pandemic, researchers found that participants with healthcare workers at home had stronger self-efficacy than others (34).

Moreover, the idea that self-efficacy can directly and indirectly affect mental health is also supported by research findings. Arabian et al. (35) demonstrated that self-efficacy can improve individual mental health. Lei et al. (36) found that individuals with low general self-efficacy tend to focus on their own shortcomings and are more likely to show emotional reactions, such as anxiety and depression. However, individuals with high general self-efficacy tend to be more willing to accept challenges and show more active and positive emotional responses by constantly improving their ability to cope with difficulties. Additionally, self-efficacy can encourage individuals to maintain healthy behaviors, so as to maintain psychological stability (37). Ievers-Landis et al. (30) found that self-efficacy plays a partial mediating role in the association between family support and calcium intake to prevent osteoporosis. Further, individuals with high self-efficacy may experience more positive outcomes from help-seeking behavior. Therefore, the psychological cost of seeking help is lower and, in turn, people will actively seek help to relieve stress and maintain, or even improve, their physical and mental health. A cross-sectional study of 250 individuals showed self-efficacy working as a mediator in the relationship between social support and serious mental illness recovery (38). Therefore, Hypothesis 3 was proposed as follows: self-efficacy plays a mediating role in the relationship between social support and mental health.

### Moderating Effects of Risk Level

The mental model of risk proposed by Svenson (39) describes individuals' factual cognition of the contingency formed by risk events and their overall value judgment. The threshold of people's risk acceptability is closely related to their potential reactions, while a single risk event with strong signal value may cause risk amplification. When the risk exceeds a level that an individual finds acceptable, he or she will show a strong reaction, which will lead to aggravation of difficulty in risk communication, and psychological reactions such as anxiety and panic, which will harm one's mental health (39, 40). COVID-19 is highly infectious, current scientific understanding of the coronavirus is insufficient, and the treatment of COVID-19 lacks targeted and efficient medical methods. It can be said that COVID-19 is a huge disaster for all of society and even for all humankind. Therefore, in this study, risk level was defined as whether there were confirmed/suspected cases in one's vicinity (workplace or home, including residents in the same community).

Because COVID-19 is highly contagious, there is a significant risk that other people will be infected if there is a confirmed or suspected case nearby. For community residents, although the “home quarantine order” objectively reduces people's risk of infection and protects people's lives to a large extent, due to the high-risk characteristics of COVID-19 itself, people's subjective panic about COVID-19 may persist, and their mental health will remain threatened. Moreover, according to previous psychological research during the SARS outbreak, individuals without direct experience were vulnerable to the influence of geographical and media information factors, resulting in psychological reactions, such as anxiety and panic, toward SARS. Particularly, when relevant information did not provide clear guidance, individuals were found to be prone to have adverse psychological reactions that endangered their mental health (40, 41). Therefore, whether there are confirmed or suspected cases within one's proximity is a specific and direct source of risk, and will have an impact on individual mental health.

However, due to differences in experience, ability, and knowledge, different groups may construct different psychological patterns, and these differences can affect people's ability and willingness of risk-acceptance (39). Studies have shown that under COVID-19, the self-efficacy of individuals with advanced educations and with medical and nursing workers at home (34) is higher than that of those without medical and nursing workers in the family or with a low-level educational background. Thus, it can be speculated that experience—including indirect experience provided by family members working in healthcare fields—and knowledge give individuals more psychological energy (self-efficacy), which may make them more receptive to risks and less likely to have their mental health impacted. However, according to research conducted during the SARS epidemic, both healthcare workers and residents in affected areas experienced certain levels of stress, and even panic, anxiety, and other adverse psychological reactions, resulting in impaired physical and mental health (42). Therefore, when the risk level is high, the maintenance/promotion effect of self-efficacy on mental health appears to be weakened. Therefore, Hypothesis 4 was proposed as follows: risk level moderates the relationship between self-efficacy and mental health.

## Materials and Methods

### Samples and Procedures

In this study, 16,616 questionnaires were collected online. Of those, 1,551 questionnaires for healthcare workers were deleted, and 1,554 invalid questionnaires that were answered in <200 s, or the respondents were than 16 or more than 100 years old, were also deleted. Finally, 13,511 valid questionnaires were included for analysis (response rate = 81.3%). The sample included respondents from all 18 cities in Henan province, China. Among them, there were 4,267 men (31.6%) and 9,244 women (68.4%). Mean participant age was 32.10 (± 11.11) years, with an age range of 16–77 years. Among the participants, 2,930 (21.7%) had a high school education, 2,761 (20.4%) had a junior college education, and 7,820 (57.9%) had a bachelor's degree or above. Additionally, 1,900 (14.1%) had healthcare workers in their families, while 11,611 (85.9%) had no healthcare workers in their families.

In the present study, the convenient sampling (snowball sampling) method were conducted to collect data from 17:00 Jan 27th to 17:00 Jan 29th, during the growing period of the pandemic in China. The online platform we used to upload the questionnaire named wjx, which is enpowered by www.wjx.cn. It is the largest and most widely used questionnaire survey platform in China that provides functions equivalent to Amazon Mechanical Turk. The questionnaire was uploaded to the platform, which automatically generates a network link. The link was then posted via the researcher's social media account and the organization's website, inviting people to answer the questions and forward the questionnaire of their own accord. It is totally anonymous, and participants were told that they can withdraw at any time they want in the instructions. This is an unpaid public interest survey, and in both the instruction and the conclusion of the questionnaire, we asked participants if they would like to forward the questionnaire to others.

### Measures

A self-compiled COVID-19 Social Mentality Questionnaire was used as a measurement tool in this study. The questionnaire was prepared by psychology professors and doctoral students during the early stage of the COVID-19 pandemic, after referring to previous studies of the SARS epidemic and relevant literature on sudden public health events. Based on important documents and public voices during the COVID-19 pandemic, this measurement tool was designed to investigate public mentality during the pandemic based on seven aspects: (1) the cognition of the COVID-19 pandemic; (2) knowledge of how to prevent COVID-19; (3) physical and mental symptoms of COVID-19 patients and the public; (4) the public's irrational behaviors during the COVID-19 pandemic; (5) the public's need for psychological assistance; (6) the public's self-efficacy in seeking help during the COVID-19 pandemic; and (7) the public's interest behaviors (intention) during the COVID-19 pandemic. After determining the basic framework, the team members modified and improved the questionnaire items through several discussions, and screened and integrated similar questions. After standardizing and modifying the content, expression, and format of the first draft of the questionnaire, the final draft was completed. Then, the questionnaire was uploaded to an online platform, and psychology scholars and postgraduates were invited to participate in a pilot test. The questionnaire was refined according to their feedback, and finally, the formal questionnaire was completed. The formal questionnaire was then uploaded to an online platform, where it was distributed within a wide-ranging population.

#### Mental Health

In the present study, respondents' mental health was measured by a self-report question: “In general, how do you feel about your mental health?” Answers choices were “better than usual,” “as usual,” and “worse than usual.” Those who chose “better than usual” or “as usual” were considered to be mentally healthy, and their score was “1.” Those who chose “worse than usual” were considered to be in poor mental health, and their score was “0.”

#### Social Support

Social support was measured by a self-report question: “Is someone in your family a healthcare worker?” The answer “yes” was scored as “1,” and the answer “no” was scored as “0.”

#### Pandemic-Related Knowledge

The sub-scale “Cognition Questionnaire on COVID-19 Pandemic” from the self-compiled COVID-19 Social Mentality Questionnaire was used to measure respondents' pandemic-related knowledge. The questionnaire consists of eight items, which, respectively, examine the participants' cognition on the characteristics of COVID-19 infection, main symptoms, route of transmission, knowledge of prevention and the difference between its symptoms and those of the common cold/flu, and research progress related to the disease and development stage of the pandemic (see Appendix 1). Total scores range from 0 to 8; answers of “very unclear” and “relatively unclear” are scored as “0,” and answers of “very clear” and “relatively clear” are scored as “1.” Cronbach's alpha for this questionnaire was 0.697.

#### Self-Efficacy

The sub-questionnaire “The Public's Self-Efficacy in Seeking Help During the COVID-19 Pandemic” from the self-compiled COVID-19 Social Mentality Questionnaire was used to measure respondents' self-efficacy. It includes four items, which, respectively, examine participants' information acquisition efficacy, information identification efficacy, medical treatment acquisition efficacy, and psychological assistance acquisition efficacy (see Appendix 1). Answers of “yes” are scored as “1,” and answers of “no” or “uncertain” are scored as “0,” for a total score ranging from 0 to 4. Cronbach's alpha for this questionnaire was 0.750.

#### Risk Level

Risk level was evaluated by a single self-report question: “Are there confirmed or suspected cases in your area?” Answers of “yes” were scored as “1,” and answers of “no” were scored as “0.”

### Data Analysis

SPSS 25.0 (IBM, Armonk, NY) was used to analyze the collected data. Descriptive analysis was used to describe participants' mental health profiles and other study variables. Pearson's test was applied to examine correlations among the variables. A hierarchical regression analysis was conducted to investigate the mediating effect of pandemic-related knowledge in the relationship between social support and self-efficacy, the mediating effect of self-efficacy in the relationship between social support and mental health, and the moderating effect of risk level in the relationship between self-efficacy and mental health.

## Results

### Mental Health Profile

Overall, 1,450 (10.7%) participants rated their mental health as “worse than usual” during the pandemic, 11,649 (86.2%) rated it as “usual,” and 412 (3.0%) rated it as “better than usual.”

### Variables Correlations

The descriptive statistics and correlation matrices of each research variable are shown in Table 1. As can be seen from Table 1, there were significant positive correlations between social support and risk level (*r* = 0.045, *p* < 0.01), pandemic-related knowledge (*r* = 0.043, *p* < 0.01), and self-efficacy (*r* = 0.034, *p* < 0.01); however, no significant correlation was found between social support and mental health (*r* = −0.006, *p* = 0.52 > 0.05). Pandemic-related knowledge was positively correlated with self-efficacy (*r* = 0.298, *p* < 0.01) and mental health (*r* = 0.042, *p* < 0.01). There was a significant positive correlation between self-efficacy and mental health (*r* = 0.149, *p* < 0.01). Risk level was negatively correlated with pandemic-related knowledge (*r* = −0.032, *p* < 0.01), self-efficacy (*r* = −0.043, *p* < 0.01), and mental health (*r* = −0.061, *p* < 0.01).

**Table 1:** Descriptive statistics and correlations among the variables (*N* = 13,511).

**Variables**	**M ± SD**	**1**	**2**	**3**	**4**	**5**	**6**	**7**	**8**
1. Gender	0.32 ± 0.31	1.000							
2. Age	32.08 ± 11.09	0.039^**^	1.000						
3. Education	1.36 ± 0.82	−0.049^**^	−0.102^**^	1.000					
4. Social support	0.14 ± 0.35	−0.009	0.019^*^	0.111^**^	1.000				
5. Risk level	0.05 ± 0.22	−0.010	−0.078^**^	0.060^**^	0.045^**^	1.000			
6. Pandemic-related knowledge	6.61 ± 1.39	−0.040^**^	0.035^**^	0.099^**^	0.043^**^	−0.032^**^	1.000		
7. Self-efficacy	2.85 ± 1.34	0.093^**^	0.018^*^	0.012	0.034^**^	−0.043^**^	0.298^**^	1.000	
8. Mental health	0.89 ± 0.31	0.038^**^	−0.017^*^	−0.022^*^	−0.006	−0.061^**^	0.042^**^	0.149^**^	1.000

* *P < 0.05*,

** *P < 0.01*.

### Test of the Moderated Mediation Model

**Figure 2:**
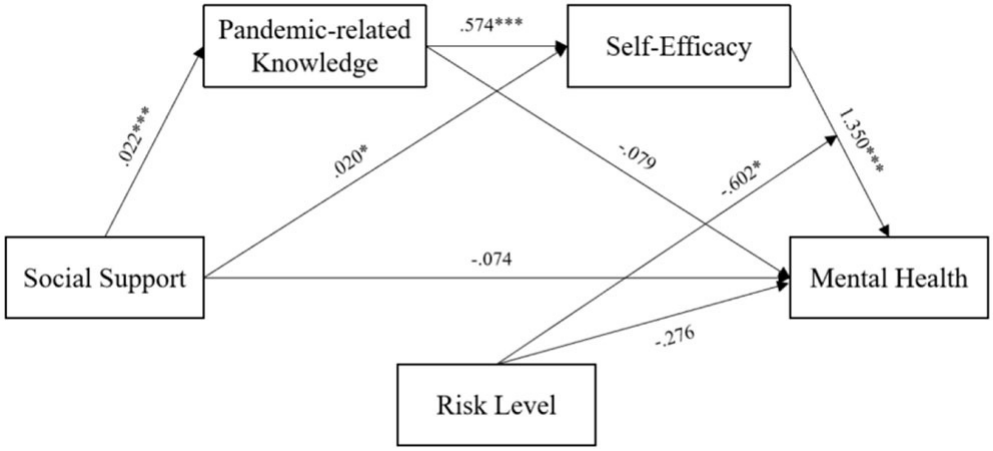
The moderated mediation model. Pandemic-related knowledge mediated the relationship between social support and self-efficacy, self-efficacy mediated the relationship between social support and mental health, and the risk level moderated the relationship between self-efficacy and mental health. *P < 0.05, ***P < 0.001.

The results of the moderated mediation model testing method recommended by Wen and Ye (43) are shown in Table 2. In Equation (1), social support had a significant positive predictive effect on pandemic-related knowledge (β = 0.022, *t* = 5.021, *p* < 0.001), which indicated that individuals who have family members in the healthcare industry receive more knowledge/information about the pandemic. In Equation (2), social support had a significant positive predictive effect on self-efficacy (β = 0.020, *t* = 2.524, *p* = 0.012 < 0.05), indicating that the higher a participants' level of social support, the higher his or her level of self-efficacy. Additionally, pandemic-related knowledge had a significant positive predictive effect on self-efficacy (β = 0.574, *t* = 36.212, *p* < 0.001), which indicated that the more pandemic-related knowledge a participant acquired, the higher his or her self-efficacy would be. Therefore, pandemic-related knowledge was found to play a partial mediating role in the relationship between social support and self-efficacy. In Equation (3), social support (β = −0.074, *Z* = −0.929, *p* = 0.353 > 0.05) and pandemic-related knowledge (β = −0.079, *Z* = −0.497, *p* = 0.619 > 0.05) had no significant predictive effect on mental health, while self-efficacy had a significant positive predictive effect on mental health (β = 1.350, *Z* = 16.065, *p* < 0.001). This indicated that self-efficacy has a fully mediating role in the relationship between social support and pandemic-related knowledge and mental health. In Equation (3), the interaction between self-efficacy and risk level had a significant negative predictive effect on mental health (β = −0.602, Z = −2.133, *p* < 0.05). Therefore, risk level was found to have a moderating effect on the relationship between self-efficacy and mental health, which constituted a moderated mediation model. Based on the above results, the moderated mediation model proposed in this study was supported (see Figure 2).

**Table 2:** Test of the moderated mediation model (*N* = 13,511).

**Variables**	**Equation (1) (criterion: pandemic-related knowledge)**			**Equation (2) (criterion: self-efficacy)**			**Equation (3) (criterion: mental health)**		
	**β**	**se**	***T***	***B***	**se**	***t***	**β**	***se***	***Z***
Constant	0.823	0.002	509.680^***^	0.234	0.013	17.493^***^	1.360	0.127	10.702^***^
Social support	0.022	0.004	5.021^***^	0.020	0.008	2.524^*^	−0.074	0.080	−0.929
Pandemic-related knowledge				0.574	0.016	36.212^***^	−0.079	0.159	−0.497
Self-efficacy							1.350	0.084	16.065^***^
Risk level							−0.276	0.196	−1.409
Self-efficacy × risk level							−0.602	0.282	−2.133^*^
R^2^	0.002			0.090			0.047		
F	25.214^***^			664.008^***^					
-2LL							8892.164^***^		

* *P < 0.05*,

*** *P < 0.001*.

Omnibus tests were used to examine the integration of the regression model ratio. The likelihood ratio test results (χ^2^ = 318.964, *p* < 0.001) of all model parameters indicated that among the variables included in the fitted model, the OR value of at least one variable was statistically significant; that is, the overall model was significant. The Hosmer–Lemeshow Test was used to test the goodness of fit of the regression model, and the results (χ^2^ = 4.959, *p* =0.175 > 0.05) showed that the information in the current data had been fully extracted, and the goodness of fit of the model met the requirements.

To further analyze the size and confidence interval of the moderated mediation model effect, a total of 13,511 samples with 1,000 iterations were conducted in the conditional indirect effect test program developed by Preacher et al. (44). According to the No. 87 model in PROCESS, the parameters were estimated with the bias-corrected non-parametric percentile Bootstrap method, and the results are shown in Table 3. When the risk level was 0, the mediating effect of self-efficacy was 1.350, accounting for 64.3% of the total effect. This suggested that individuals with high self-efficacy are more likely to maintain or improve their mental health during the pandemic when risk levels are low. When the risk level was 1, the mediating effect of self-efficacy was 0.748, accounting for 35.7% of the total effect. This indicated that during the pandemic, when the risk level is high, the promotion effect of self-efficacy on mental health will be weakened.

**Table 3:** Mediating effects and confidence intervals at different levels of the moderating variable (*N* =13,511).

**Risk level**	**Effect**	***SE***	**Bootstrap (95% CI)**
0.000	1.350	0.084	(1.185, 1.515)
1.000	0.748	0.272	(0.215, 1.281)

## Discussion

The results of the present study showed that during the COVID-19 pandemic, the majority of participants rated their mental health status as usual; however, 10.7% reported that their mental health had declined due to the pandemic. The result is consisted with the previous study that Wang and Li (45) found that only 6.8% of participants claimed that they have sleep problems during the COVID-19 pandemic. However, it is noted that there are large data differences among studies by different scholars, especially those of samples from different countries. Staines (20) and Killgore et al. (21) reported that during the COVID-19 pandemic, 43% of the English-speaking U.S. adults suffered loneliness and 56% of them had sleep difficulty, which finally resulted in mental health decline, and even suicide ideation rise. The differences between the two countries may attributed to the following reasons. First, the home quarantine order in China happens to be during the Spring Festival holiday, which is a time to get together with family members and get family comfort. Most people have been reunited with their families before the home quarantine order begin, thus, individuals are less likely to feel being lonely. Second, the successful experience of the Chinese people in overcoming SARS in 2003 may strengthen their confidence in overcoming the COVID-19 pandemic.

Besides, although the results of this paper show that 10.7% of the participants have a decline in mental health, considering China's huge population base and limited psychological assistance ability, 10.7% is not a small proportion, and it may bring great challenges to the social psychological service system and the stability of society. Therefore, it is necessary to pay attention to public mental health. When sharing knowledge and prevention methods for the coronavirus with the public, it is also necessary to include knowledge of mental health protection, so as to use scientific and professional knowledge to prevent the public's mental health decline. Furthermore, it is suggested that more professional psychological resources should be devoted to the prevention and control of the pandemic, and more information on the psychological change process and effective coping measures should be provided to the public. Additionally, more psychological assistance hotlines should be opened to allow people more access to mental health assistance and create a positive and healthy social psychological atmosphere.

The findings further demonstrated that social support is not a direct predictor of mental health. According to the research of Chinese scholars Xiao and Yang (13), social support is divided into objective support, subjective support and support utilization, which are not completely consistent with the correlation or predictive role of mental health. A meta-analysis of social support using Chinese academic papers also showed that the objective support dimension was slightly positively correlated with the total mental health score, while the subjective support dimension and the utilization dimension of support were moderately negatively correlated with the total mental health score, while objective support was negatively correlated with depression, anxiety, compulsion, somatic symptoms and other factors in SCL-90 (19). In addition, some research results show that basing on different operational definitions (such as subjective support, overall social support, etc.), social support has a small/moderate negative correlation with mental health (19). Since the operational definition of social support in this study was defined as family-based social support that is different from that used in previous studies, it was considered as a resource in the present study. However, considering the social support was measured by the question of “whether someone in the family is a healthcare worker,” in this study, social support actually refers to objective support based on the family. Nevertheless, whether the role of this support can work and how effect its role is depends to a large extent on participants' subjective perception of objective support and support utilization. The results of this study show that self-efficacy is an important mediator between social support and mental health (two paths). In terms of the definition of self-efficacy, it is an individual's subjective evaluation of his/her effectiveness in coping with the pandemic, which is based on the subjective perception of objective support. Therefore, although social support was not found to directly predict mental health, they are still closely related, and social support indirectly affects mental health. Besides, in the current context, healthcare workers are on the frontlines in the fight against the pandemic, and COVID-19 is highly contagious. People's concerns regarding family members who are healthcare workers may create feelings of anxiety, which can negatively impact mental health.

Furthermore, social support can maintain/improve mental health through pandemic-related knowledge and self-efficacy. The results of the present study supported a partial mediating effect of pandemic-related knowledge on the relationship between social support and self-efficacy, and the complete mediating effect of self-efficacy on the relationship between social support and mental health. Social support can directly increase people's self-efficacy, and can also promote self-efficacy by improving pandemic-related knowledge, so as to maintain and even improve individual mental health during major pandemics. Precisely, individuals who have family members in the healthcare industry have more opportunities to acquire more information about the pandemic. By this way, their self-efficacy would be fostered to benefit their mental health when facing with serious crisis events. This is consistent with previous research results (26, 46, 47). During the SARS epidemic, the public's lack of knowledge about SARS led to them to experience panic; however, as related information became clear, the epidemic's impact on public mental health gradually weakened (46). Moreover, the better the public's awareness was regarding knowledge of and preventive measures for SARS, the less possibility of them to show symptoms of mental health issues/disorders (47). Chen et al.'s (26) survey on the public's mentality during the COVID-19 outbreak also indicated that the clearer the public's understanding of the pandemic and the progress of COVID-19 research, the less fluctuations there were in public mental health indicators. Therefore, public awareness of COVID-19 knowledge should be strengthened, and information about the epidemic should be released quickly, accurately, and transparently. Not only could this promote the public's understanding of COVID-19, but it would also lessen their anxiety and panic. Moreover, it is also conducive to mobilizing society as a whole to take coordinated actions and participate in pandemic control.

Nevertheless, the complete mediating effect of self-efficacy indicated that the positive effects of pandemic-related knowledge and social support on mental health are realized by improving individual self-efficacy. Social cognition theory posits that self-efficacy is a cognitive factor, and individual cognition can effectively regulate thoughts and behaviors (27). Previous studies have reached similar conclusions. Zhao and Wang (34) investigated self-efficacy during the COVID-19 pandemic, and found that the family members of healthcare workers affected by positive spillovers tended to have higher self-efficacy, as did individuals with higher knowledge reserves. According to previous studies (19), under normal circumstances, relatively disadvantaged groups, such as older adults and students, have a higher need for social support and channels of knowledge and information. Thus, in the face of the raging COVID-19 pandemic, people's overall self-efficacy, compared with normal situations, may decline, and most people could become “relatively disadvantaged,” Thus, people will need more social support and sources of knowledge than usual, to maintain their self-efficacy at the normal level. Therefore, people need to show more cooperation and solitary, to support each other and maintain or even improve their mental health, which is more conducive to containing the pandemic, reducing the death toll, and finally defeating COVID-19.

The results also showed that risk level had a moderating effect on the relationship between self-efficacy and mental health; thus, at a high risk level, the role of self-efficacy in maintaining and promoting mental health would be weakened. According to the mental model of risk (39), when individuals face a risk that exceeds their ability and willingness to accept the risk, they may have a strong physical and mental reaction, such as panic and anxiety. Moreover, for individuals, the threat of risk decreases with the increase of geographical distance (48). Thus, when people learn of a confirmed or suspected case nearby, it means they are geographically close to danger, and their sensitivity to the risk will naturally increase. Moreover, since scientists still do not fully understand the novel coronavirus, and there is currently no specific confirmed treatment, the risks posed by the virus are far beyond an individual's control. According to control theory (49), when individuals cannot correctly identify the source of a threat, and do not know which methods and information can effectively protect them, they will feel a loss of control and experience stress, which leads to simple and crude one-sided interpretations of the threat. However, these one-sided interpretations cannot bring meaningful psychological comfort, and will lead to cognitive dissonance, thus further aggravating one's sense of losing control, and increase anxiety and panic. Further, the chaos that accompanies the sense of losing control may be more harmful than the disease itself.

Presently, the pandemic is still a serious threat, and governments should take aggressive prevention and control measures that respect science, focus on quality allocation of resources, and make every effort to reduce the risk level. Further, healthcare authorities should work with psychological support agencies and other industries (e.g., internet industry, news communication industry) to ensure that information channels are fully open and effective, so that everyone can clearly understand how to identify accurate information and help themselves and others. When people have high levels of self-efficacy, they will have more strength and confidence to overcome the circumstances created by the pandemic. Additionally, pandemic prevention and control is related not only to personal safety and health but also to regional stability and the development of the global economy. Therefore, all people should cooperate with each other to realize the optimal allocation of resources, improve the utilization of resources, solve problems, and achieve victory over COVID-19 as soon as possible.

### Limitations

First, due to the limitations of the current situation, this study adopted convenient sampling; therefore, the participants could not fully represent the general population, and the generalization of the results is limited. In future studies, it is recommended that researchers adopt a more representative sampling method, and conduct sampling in a wider area, to increase the generalizability of the results. Second, due to the sudden and unpredictable nature of the COVID-19 outbreak, the social mentality questionnaire used in this study still requires improvement. It is expected that in future studies, researchers can design more accurate measurement tools to study social mentality in major pandemics, according to research needs. Third, this study used a cross-sectional design to investigate public mental health and influencing factors within a limited time period. It was impossible to make a longitudinal comparison of people's mental health status and its influencing factors at different stages of the pandemic and conduct a comprehensive investigation. Therefore, it is suggested that researchers should investigate more variables that may affect mental health, and combine multiple research designs to conduct a comprehensive and in-depth study of people's mentality and behaviors during the pandemic.

### Conclusions

This study revealed the important impact of social support, pandemic-related knowledge, self-efficacy, and risk on mental health during a major pandemic. In the face of the novel coronavirus, encouragement and support between people can help to promote the transmission of knowledge and information and enhance self-efficacy, so as to maintain physical and mental health. By extension, solidarity and cooperation between countries and regions will help overcome COVID-19 faster and more effectively, and safeguard the health of all humankind. Further, in the face of a major pandemic, aggressive science-based government policies are a key factor to effectively improving people's confidence and reducing external risks. The healthcare workers who are fighting against the pandemic need more encouragement and support from society as a whole.

Hats off to the people who protect and support us during COVID-19 pandemic.

## Data Availability Statement

The raw data supporting the conclusions of this article will be made available by the authors, without undue reservation.

## Ethics Statement

The studies involving human participants were reviewed and approved by Henan University Institutional Review Board. Written informed consent to participate in this study was provided by the participants' legal guardian/next of kin.

## Author Contributions

YL and SW are the principal investigators for the study, generated the idea, and designed the study. SW and KF were the primary writers of the manuscript and approved all changes. SW, KF, and YZ supported the data input and data analysis. JL and WW supported the data collection. All authors were involved in developing, editing, reviewing, and providing feedback for this manuscript and have given approval of the final version to be published.

## Conflict of Interest

The authors declare that the research was conducted in the absence of any commercial or financial relationships that could be construed as a potential conflict of interest.

## Appendix 1

The 15 items involved in this study are listed below:

**Social Support:** “Is someone in your family a healthcare worker?”

**Mental Health:** “In general, how do you feel about your mental health?”

**Risk Level:** “Are there confirmed or suspected cases in your area?”

**Pandemic-related Knowledge:**

a) Do you know the main symptoms of COVID-19?
b) Do you know how COVID-19 is transmitted?
c) Do you know the difference in symptoms between COVID-19 and the common cold?
d) Are you aware of the current pandemic?
e) Are you aware of current research progress on COVID-19?
f) Do you think wearing a mask can prevent COVID-19 infection?
g) Do you know how to wash your hands properly?
h) Do you think that the behaviors of dining and gathering is at risk of COVID-19 infection?

**Self-efficacy:**

a) I am sure I have the resources I can use to gain knowledge about COVID-19.
b) I'm sure I know how to distinguish the rumor from the truth.
c) I'm sure I know how to get proper medical treatment if I need it.
d) I'm sure I know how to get the proper psychological services if I need it.
